# Dysfunctional HDL and progression of atherosclerosis in HIV-1-infected and -uninfected adults

**DOI:** 10.1186/1476-511X-12-23

**Published:** 2013-03-05

**Authors:** Theodoros Kelesidis, Otto O Yang, Michelle A Kendall, Howard N Hodis, Judith S Currier

**Affiliations:** 1 Department of Medicine, Division of Infectious Diseases, David Geffen School of Medicine, University of California, Los Angeles, CA, USA; 2 Department of Microbiology, Immunology, and Molecular Genetics, David Geffen School of Medicine, University of California, Los Angeles, CA, USA; 3 Center for Biostatistics in AIDS Research, Harvard School of Public Health, Boston, MA, USA; 4 Atherosclerosis Research Unit, Department of Medicine and Preventive Medicine, Keck School of Medicine, University of Southern California, Los Angeles, CA, USA; 5 Center for Clinical AIDS Research and Education, David Geffen School of Medicine, UCLA, 9911 W. Pico, Suite 980, Los Angeles, CA 90035, USA

**Keywords:** HIV-1, High density lipoprotein (HDL), HDL function, Dysfunctional HDL, Redox activity, Atherosclerosis

## Abstract

**Background:**

HDL function rather than absolute level may be a more accurate indicator for risk of developing atherosclerosis. Dysfunctional HDL has increased redox activity and reduced antioxidant properties, but it is unknown whether abnormal HDL function is associated with progression of atherosclerosis in HIV-1-infected subjects.

**Findings:**

We retrospectively measured serum HDL function in 91 subjects from a prospective 3-year study of carotid artery intima-media thickness (CIMT), which enrolled triads of risk factor-matched persons that were HIV-1-uninfected (n=36) or HIV-1+ with (n=29) or without (n=26) protease inhibitor (PI)-based therapy for ≥ 2 years. HDL function was assessed using a biochemical assay that measures the oxidation of dihydrorhodamine 123 (DHR oxidation rate, DOR), in which higher DOR readout corresponds to dysfunctional HDL phenotype.

There were no significant associations between DOR and HIV-1 infection. In univariate analysis of 55 HIV-1-infected subjects, greater waist circumference and lower serum HDL were significantly associated with higher baseline levels of DOR (p=0.01). These subjects had significant increases in levels of DOR over time (3 years) that were associated with white race (p=0.03), higher nadir CD4 count (p<0.001), and lower baseline CIMT (p<0.001). Lower baseline HDL levels, but not function of HDL (p>0.1) (DOR), were significantly associated (p=0.02) with progression of CIMT.

**Conclusion:**

In a small matched cohort study of HIV-1-infected subjects who had a low cardiovascular risk profile, HDL function changed over time and was independently associated with anthropometric parameters of obesity but not with progression of CIMT.

## Findings

### Introduction

High-density lipoprotein (HDL) function rather than absolute level may be a more accurate indicator for risk of developing atherosclerosis [1]. Systemic inflammation may involve the conversion of HDL to a dysfunctional form that is no longer cardioprotective and contributes to the risk of coronary disease [2,3]. Dysfunctional HDL has increased redox activity [4] and reduced antioxidant properties that may contribute to atherogenesis [5,6]. We have recently shown that HIV-1-infected (HIV-1+) subjects have dysfunctional HDL [4,7,8]. However, no study has directly explored the relationships between dysfunctional HDL, HIV-1 infection and progression of atherosclerosis as well as predictors of dysfunctional HDL in HIV-1 infection.

Using a recently developed biochemical assay that quantifies the redox activity of HDL based on the oxidation rate of the fluorogenic probe dihydrorhodamine 123 (DOR) [4] and serum specimens from a previously reported clinical study, we examined the longitudinal association of DOR with the progression of atherosclerosis, as evaluated by measurement of carotid artery intima-media thickness (CIMT), in HIV-1-infected (HIV-1+) and -uninfected (controls) individuals. Since residual immune activation despite antiretroviral treatment may contribute to the presence of dysfunctional HDL in HIV-1+ persons [8] and progression of atherosclerosis [9], we also investigated possible associations between DOR and biomarkers of macrophage activation, such as soluble CD14 (sCD14) and biomarkers of bacterial translocation such as lipopolysaccharide (LPS) [10].

### Methods

The current study is a subset analysis of samples obtained from a prospective, matched cohort study [9] in which subjects were enrolled as risk factor-matched triads of HIV-1+ individuals with viremia < 500 RNA copies/ml with (n=29) or without (n=26) use of PI therapy, and HIV-1-uninfected (control) individuals (n=36) from 41 triads.

Presence of metabolic syndrome, CIMT and baseline variables (fasting glucose, lipids, insulin, cardiovascular disease-related measurements, CD4^+^ T cell counts and HIV RNA levels, high-sensitivity C-reactive protein, serum sCD14, serum LPS) have previously been determined [9]. Stored samples from baseline and week 144 (or 96 if week 144 was missing) were assayed. HDL was isolated using ultracentrifugation and the DOR of each sample was determined as previously [4] and was normalized (nDOR) as ratio to the DOR of a control HDL isolated from pooled serum from healthy subjects (see Additional file 1).

Matched analyses comparing the combined HIV-1+ group to the controls (N=32) assessed the effect of HIV-1 infection on nDOR. Progression of atherosclerosis was evaluated as yearly rate of change in CIMT (ΔCIMT); and CIMT progression (previously defined as yearly rate of change ≥ 12.2 μm/yr) [9]. Mixed models regression with triad as a random effect and repeated measures regression evaluated associations of baseline measurements of nDOR and yearly rates of change in the outcome, respectively, with baseline covariates. SAS 9.2 (SAS Institute Inc., Cary, NC, USA) was used for all statistical analyses.

## Results

### HIV-1+ subjects had more metabolic abnormalities than the control subjects

As reported previously the HIV-1+ subjects in this cohort had significantly (p<0.05) higher presence of metabolic syndrome, higher levels of total cholesterol, triglycerides and non-HDL cholesterol and insulin levels, serum sCD14 and similar levels of serum LPS compared to the control subjects [9]. Due to matching the groups were similar with respect to age, sex, race, body mass index (BMI), LDL and HDL levels [9].

### HIV-1 infection was not significantly associated with baseline HDL function, but HDL function changed significantly over time within the HIV-1+ subjects

In view of our previous findings that HIV-1+ subjects have dysfunctional HDL [4,7,8], we investigated possible associations between treated HIV-1 infection and changes in serum nDOR. As shown in Table 1, at baseline and week 96/144, serum nDOR were not significantly different between both HIV-1+ groups (with and without PI treatment) and in the combined HIV-1+ group compared to the controls (p>0.2). Although the HIV/non-PI group had a significant increase in relative median levels of nDOR of 0.17 (p=0.01) by 96/144 weeks compared to baseline, there was a trend towards significant increases in nDOR over time (yearly rate of change in nDOR or ΔnDOR) within the combined HIV-1+ group (p=0.08); this was not seen within the HIV/PI and not HIV groups (p>0.3).

**Table 1:** **Summary of normalized to control (pooled HDL sample from all study subjects) DOR (nDOR) results [Median (IQR)] by group and of parameters that were significantly associated with nDOR and normalized Yearly Rate of Change in nDOR (ΔnDOR) (see Table**2**)**

**Characteristic**	**Total (N=91)**	**HIV (N=55)**	**HIV/PI (N=29)**	**HIV/non-PI (N=26)**	**Not HIV (N=36)**	**P value**
Baseline nDOR	1.64 (1.47, 2.02)	1.62 (1.44, 1.96)	1.60 (1.46, 1.95)	1.65 (1.44, 1.96)	1.65 (1.51, 2.07)	0.41^1^; 0.70^2^
Week 96/144 nDOR	1.80 (1.50, 2.00)	1.80 (1.45, 1.92)	1.79 (1.45, 1.83)	1.82 (1.45, 2.29)	1.82 (1.53, 2.14)	0.36^1^;0.27^2^
Yearly rate of change in nDOR	0.009 (-0.055, 0.108)	0.005 (-0.048, 0.110)	-0.004 (-0.055, 0.101)	0.059 (-0.031, 0.118)	0.009 (-0.059, 0.102)	0.94; 0.58; 0.29; 0.65^3^
P Value for ΔnDOR within group ^4^	-	0.08	0.92	0.01	0.31	-
Sex (Male)	84 (92%)	52 (95%)	28 (97%)	24 (92%)	32 (89%)	0.43^1^; 0.55^5^
White non-Hispanic race	69 (76%)	42 (76%)	23 (79%)	19 (73%)	27 (75%)	0.62^1^; 0.40^5^
Body mass index (kg/m^2^)	24.70 (23.40-27.60)	24.60 (23.20-27.40)	25.50 (23.60-27.60)	24.20 (22.00-26.60)	25.00 (23.75-27.95)	0.55^1^; 0.25^2^
Waist circumference (cm) ≥ 90 ^6^	40 (44%)	24 (44%)	17 (59%)	7 (27%)	16 (44%)	1.0^1^; 0.05^5^
Waist/Hip ratio ^6^	0.91 (0.86-0.94)	0.92 (0.88-0.95)	0.93 (0.92-0.96)	0.90 (0.86-0.94)	0.89 (0.83-0.92)	0.006^1^; 0.003^2^
Baseline CIMT (μm)	707.5 (640.0-767.0)	715.0 (640.0-771.0)	752.0 (631.0-778.5)	701.3 (669.5-760.5)	684.0 (639.5-758.0)	0.48^1^, 0.56^2^
Nadir CD4+ T-cells ≤ 200 (cells/mm^3^) ^7^	(-)	20 (36%)	11 (39%)	9 (35%)	(-)	0.78^5^
HDL Cholesterol < 35 (mg/dl)	20 (22%)	13 (24%)	7 (24%)	6 (23%)	7 (19%)	0.64^1^; 0.90^5^
Insulin mU/l ^8^	6.25 (5-8)	6.50 (5.10-10.70)	7.40 (6.20-14.50)	5.55 (4.55-7.15)	6 (5-7.60)	0.16^1^; 0.004^2^
LPS (pg/ml)	453.2 (243.5-795.5)	439.4 (207.1-719.5)	453.2 (273.5-635.9)	352.5 (189.2-939.2)	496.6 (246.4-1040)	0.26^1^; 0.52^2^

Notes:

^1^ Wilcoxon test for between group differences: HIV versus not HIV.

^2^ Kruskal-Wallis test for between group differences: HIV/PI versus HIV/non-PI versus Not HIV.

^3^ Wilcoxon p-value for matched group differences within each visit week: pairings assessed were HIV versus not HIV (N=32 matched pairings), HIV/PI versus HIV/non-PI (N=18 matched pairings), HIV/PI versus not HIV (N=26 matched pairings), and HIV/non-PI versus not HIV (N=21 matched pairings).

^4^ Wilcoxon test for non-zero yearly rate of change within each group.

^5^ Fisher’s Exact test for between group differences: HIV/PI versus HIV/non-PI versus Not HIV.

^6^ Due to missing data, the sample sizes are N=90, N=28, N=26, and N=36.

^7^ Due to missing data, the sample sizes are N=53, N=27, and N=26.

^8^ Due to missing data, the sample sizes are N=84, N=27, N=24, and N=33.

### Lower baseline HDL levels and anthropometric parameters of obesity were independently associated with HDL function

It is unknown which parameters may independently predict changes in HDL function in HIV-1+ subjects. In univariate analysis of HIV-1+ subjects, HDL cholesterol <35 mg/dl (p=0.007), increased baseline waist-to-hip ratio (WHR; p=0.02), and higher baseline BMI (p=0.086) were associated with higher baseline nDOR (Table 2). In the control subjects, HDL cholesterol <35 mg/dl (p=0.03), baseline waist circumference ≥90 cm (p=0.097), and increased baseline WHR (p=0.071) were univariately associated with higher baseline nDOR. These data confirm previous findings in HIV-1-uninfected subjects that obesity and lower HDL levels are associated with dysfunctional HDL [11].

**Table 2:** Univariate associations of nDOR with baseline variables

	**HIV-1+ subjects**		**Control subjects**	
**Covariate**	**Parameter estimate (95% CI)**	**P value**	**Parameter estimate (95% CI)**	**P value**
	**Baseline nDOR (normalized ratio).**			
**HDL Cholesterol (ref: ≥35 mg/dl)**	0.357 (0.114, 0.601)	0.007	0.365 (0.029, 0.701)	0.034
**Body mass index (per 1 kg/m**^**2 **^**increase)**	0.032 (-0.005, 0.069)	0.086	(-)	(-)
**Waist circumference (ref: <90 cm)**	(-)	(-)	0.231 (-0.044,0.506)	0.097
**Waist/Hip ratio (per 0.1 unit increase)**	0.212 (0.035, 0.388)	0.022	0.215 (-0.019, 0.448)	0.071
	**Yearly rate of change in nDOR (normalized ratio/yr).**			
**Male sex**	0.156 (-0.018, 0.329)	0.078	0.067 (-0.001, 0.144)	0.085
**White vs Non-White**	0.119 (0.033, 0.206)	0.008	(-)	(-)
**Body mass index (per 1 kg/m**^**2 **^**increase)**	(-)	(-)	-0.010 (-0.020, 0.0001)	0.051
**Reported Nadir CD4 (ref: <200 cells/mm**^**3**^**)**	0.083 (0.039, 0.128)	<0.001	(-)	(-)
**Baseline CIMT (per 1 μm decrease)**	0.474 (0.229, 0.719)	<0.001	(-)	(-)
**HDL Cholesterol (ref: ≥35 mg/dl)**	-0.055 (-0.109, -0.001)	0.046	(-)	(-)
**Insulin (per 10 mU/l decrease)**	(-)	(-)	0.120 (0.056, 0.184)	<0.001
**LPS (per 100 pg/ml decrease)**	(-)	(-)	0.005 (0.002, 0.008)	0.005

The baseline variables considered for all subjects were age, gender, race, fasting lipid measurements [total cholesterol, low density lipoprotein (LDL) cholesterol, HDL cholesterol, triglycerides, and non-HDL cholesterol], use of lipid lowering drugs, fasting glucose, body mass index, waist circumference, waist/hip ratio, insulin, high-sensitivity C-reactive protein, homocysteine, sCD14, LPS, and CIMT. For the HIV-1-infected subjects, additional covariates included years of PI use, CD4+ T-cell count, and nadir CD4+ T-cell count.

### Changes in HDL function over time were associated with baseline HDL, race, nadir CD4 count, and CIMT in HIV-1+ subjects

Because there were significant changes in nDOR over time within the HIV-1+ group, we sought factors that were associated with yearly *rate* of change in HDL function (ΔnDOR) in HIV-1+ subjects. Similar to the univariate analysis (Table 2), multivariate analysis in HIV-1+ subjects showed a positive ΔnDOR was associated with lower baseline CIMT (parameter estimate 0.45, 95% CI: 0.23-0.67; p<0.001), white race (estimate 0.12, 95% CI: 0.01-0.23; p=0.03), baseline HDL ≥35 mg/dl (estimate 0.07, 95% CI: 0.02-0.12; p=0.007), and nadir CD4 >200 cells/mm^3^ (estimate 0.08, 95% CI: 0.04-0.12; p<0.001). In the control subjects, lower baseline LPS (p=0.03) and decreased baseline insulin (p=0.002) were associated with a positive ΔnDOR in multivariate analysis. There were too few non-white subjects to determine the effect of race on ΔnDOR. Finally, increased *rate* of change in nDOR in HIV-1+ subjects was associated with favorable metabolic and immunologic parameters with higher baseline HDL levels, in contrast to baseline nDOR, which was associated with lower HDL levels. Further larger prospective studies will be required to explore these findings and determine predictors of both baseline HDL function and changes in HDL function over time.

### HDL function was not associated with progression of subclinical atherosclerosis in HIV-1+ subjects

Considering the previously described role of HDL function in progression of atherosclerosis in HIV-1-uninfected subjects, we investigated the possible relationship between HDL function and progression of atherosclerosis in HIV-1+ subjects. In a prior multivariate analysis in the HIV-1+ subjects, baseline HDL <35 mg/dl (p=0.04) and higher levels of baseline sCD14 (p=0.04) and LPS (p=0.003) were associated with a positive ΔCIMT [9]. In univariate analysis in HIV-1+ subjects, baseline nDOR was not associated with baseline CIMT (estimate: 53.2; 95% CI: (-16.6-123; p=0.13), ΔCIMT (estimate: -7.5; 95% CI: (-15.6- 0.7; p=0.07), and CIMT progression (estimate: 0.8; 95% CI: 0.2-3.6; p=0.81). It is possible that different factors influence the presence of plaque and CIMT, and thus the relationship of HDL function with different measures of atherosclerosis [9] requires further examination in larger studies.

### The interaction of the HDL redox activity with levels of cholesterol may be important in HIV-1+ subjects

The redox activity of HDL may depend on the interplay between HDL and LDL and the concentrations of both HDL and LDL and their ratio (LDL/HDL) [3,4]. In HIV-1+ subjects, the baseline interaction model of nDOR*(LDL/HDL), including HDL, LDL/HDL, and nDOR*HDL, nDOR*(LDL/HDL) was significantly associated with baseline CIMT (estimate: 120; 95% CI: 15.4-225; p=0.03), but not with CIMT progression (OR: 0.5; 95% CI: 0.06-4.5; p=0.55), and ΔCIMT (estimate: -3.5; 95% CI: -14.2-7.3; p=0.52). Thus, the interaction of the HDL redox activity with levels of cholesterol may be taken into consideration when studying the association of dysfunctional HDL with atherosclerosis in HIV-1+ subjects who are known to have dyslipidemia and dysfunctional HDL [4,7,12].

### HDL redox activity was not associated with biomarkers of macrophage activation in HIV-1+ subjects

Residual immune activation despite effective antiretroviral therapy could contribute to the presence of dysfunctional HDL in HIV-1 infection [8,13,14]. However, despite the significantly higher baseline levels of sCD14, a marker of macrophage activation, in HIV-1+ compared to control subjects [10] in our small study, we did not detect any significant associations between nDOR and sCD14 (p>0.1). Larger studies are needed to elucidate the role of dysfunctional HDL in HIV-1-induced immune activation.

### Limitations/conclusion

Our study had limited statistical power to detect small differences in HDL redox activity between groups, especially in the context of the limitations of the DHR assay (see Additional file 1). Moreover, despite preliminary data from *in vitro* studies that support a role of oxidized HDL in atherosclerosis [4], the significance of the nDOR, which is a biochemical and not a physiological parameter, in studying atherosclerosis *in vivo,* needs to be validated in large-scale human studies.

Despite these limitations, our study is among the first attempts to measure HDL function in HIV-1+ and controls longitudinally and define their possible associations with progression of subclinical atherosclerosis. Thus, this study may set the basis for further studies on the role of HDL function in HIV-1 infection given that standard clinical lipid profile testing may not be an adequate measurement of the risk for atherosclerosis.

## Abbreviations

BMI: Body mass index; CI: Confidence Interval; CIMT: Carotid artery intima-media thickness; DHR: Dihydrorhodamine 123; DOR: Dihydrorhodamine 123 oxidation rate; nDOR: Normalized DOR; ΔCIMT: Yearly rate of change in CIMT; ΔnDOR: Yearly rate of change in nDOR; HDL: High Density Lipoprotein; HIV-1: Human Immunodeficiency virus-1; LDL: Low Density Lipoprotein; LPS: Lipopolysaccharide; sCD14: Soluble CD14; WHR: Waist-to-hip ratio.

## Competing interests

No competing financial interests exist. This manuscript is related to the Patent UCLA Case 2011-774-1. This work was presented partially at the XIX International AIDS Conference 2012, 22-27 July 2012, Washington, DC, USA, abstract no.THAB0201.

## Authors’ contribution

All authors contributed to the intellectual development of this work, and approved the final manuscript. TK, MK analyzed the data. TK carried out the experiments and drafted the manuscript. OY, HH, JC provided critical corrections to the manuscript.

## Supplementary Material

Additional file 1 Supplemental material. Click here for file
