# Supplementary material for: Dysfunctional HDL and progression of atherosclerosis in HIV-1-infected and -uninfected adults
Source: Lipids Health Dis. 2013 Mar 5;12:23. doi: 10.1186/1476-511X-12-23 (PMC3602051; doi:10.1186/1476-511X-12-23)

Supplemental Material

Subjects (regarding Supplemental Material)

Blood samples were collected from Human Immunodeficiency virus (HIV-1)-infected individuals on combination antiretroviral therapy with suppressed viremia (below 50 copies of RNA/ml). Normal volunteers matched by age and sex were recruited under a protocol approved by the Human Research Subject Protection Committee of the University of California, Los Angeles. Blood bank specimens were collected from healthy young blood donors according to previously well-defined criteria [1-3]. More specifically the donors were young (range 19-40 years old) had no known underlying diseases including diabetes, were known to have normal lipid profile and were not receiving hypolipidemic medications. The study was approved by the UCLA Institutional Review Board.

Reagents. Dihydrorhodamine 123 (DHR) was obtained from Molecular Probes (Eugene, OR). DHR was prepared as a stock of 50 mM in dimethyl sulfoxide (DMSO) as previously described [4,5]. Iron-free HEPES (N-2-hydroxyethylpiperazine-N'-2-ethanesulfonic acid)-buffered saline (HBS, HEPES 20 mM, NaCl 150 mM, pH 7.4) was prepared as previously described [5,6]. The DHR stock was diluted 1:1000 in HEPES saline solution to prepare a working solution of 50 µM.

HDL purification. HDL was isolated using precipitation with polyethylene glycol (PEG) and ultracentrifugation, aliquoted and stored as previously described [5]. HDL cholesterol was quantified using a standard colorimetric assay (Thermo DMA Co., San Jose, CA, USA) as previously described [7].

RHD-based cell-free assay of HDL function. Quadruplicates of HDL (2.5 µg of cholesterol unless otherwise specified) were added to 96-well plates (polypropylene, flat bottom, black,

Fisher Scientific, USA). HBS was added to each well to a final volume of 150 µl, followed by addition of 25 µl of the 50µM DHR working solution, for a total volume of 175 µl (final DHR concentration of 7 µM). Immediately following DHR addition, the plate was protected from light and placed in the fluorescence plate reader (at 37ºC). The fluorescence of each well was assessed at two minute intervals over an hour with a Synergy 2 Multi-Mode Microplate Reader (Biotek, Vermont, USA), using a 485/538 nm excitation/emission filter pair with the photomultiplier sensitivity set at medium. Determination of oxidation rate of DHR and the slope of oxidation of HDL (DOR) was performed by measuring the slope of fluorescence increase over 50 minutes after addition of a specific amount of lipid (HDL) as previously described [5]. HDL oxidative function was calculated as the mean of quadruplicates for the wells containing the HDL sample.

Pooled HDL isolated from serum from healthy subjects has a relatively narrow range of measurement of DOR and can be used as a universal control in assays that quantify HDL redox activity. The previous fluorescence based assays allow quantification of only relative differences in redox activity (expressed in arbitrary units; FU/min) between different HDL samples within the same study [8]. Lack of standardization limits comparison of results between different studies and different centers. We hypothesized that the dynamic range of measurement of DOR in healthy subjects could be reduced to a narrow range after pooling certain number of HDL samples so that the total amount of HDL cholesterol would remain stable (2.5 ug). To validate this hypothesis HDL isolated from 50 cryopreserved serum blood bank specimens was combined and pooled as described in Supplemental Figure 1 and the DOR was determined.

The redox activity of different HDL samples can be expressed as a ratio to the DOR of a control HDL isolated from pooled serum. To correct for interassay variability across different plates and to standardize the assay, a pooled HDL control sample from 30 healthy volunteers was included on each plate, DOR was determined and values for samples from subjects in the study were normalized by this pooled value. The individual normalized DOR (nDOR) [nDOR = (DOR/DOR control] is evaluated as a ratio to the DOR of a control HDL isolated from the pooled serum. Thus, using this method we avoid expression of results of DOR in arbitrary units (FU/min) and results between different studies can be comparable provided the same pooled control is used.

We then compared the range of DOR between 3 different pooled controls (each pooled control derived from 30 different blood bank samples from healthy donors) (Supplemental Figure 2). We found that the DOR value was comparable between the 3 different pooled controls (range 9445-10680 FU/min) (Supplemental Figure 2). Thus, using this methodology there was > 3-fold reduction in the variability of determination of DOR (for the same amount of HDL cholesterol) in healthy subjects. This current approach may be used to create a universal control for determination of DOR by combining HDL samples from at least 30 different donors.

Limitations of Fluorescence-based biochemical assays that measure oxidative properties of HDL

Both the rhodamine assay and a previously described assay that was based on conversion of dichlorodihydrofluorescein diacetate (DCF-DA) to fluorescent DCF (2',7'-dichlorofluorescein) [7] have several limitations. We exclusively used in vitro generated oxidized forms of HDL,

whereas the oxidative modifications occurring to HDL in the diseased artery wall are conceivably more complex [9]. An additional potential limitation of the present assay is that HDL is subject to continuous remodeling in vivo [10].

In addition the magnitude of the differences in HDL redox activity between different patients may be low based on preliminary small pilot studies [5]. Thus, it is possible that the true differences in DOR between groups are smaller and, therefore, would require a larger sample size to effectively quantify relative differences in DOR between samples. Ongoing large studies from our group may address this research question.

Another limitation of the assay is that the complexity of the lipid-probe-ROS interactions of apolipoprotein (apo)B-depleted serum in the setting of systemic oxidation or inflammation may complicate interpretation of the DOR results [5]. Although we have recently determined lipid probe interactions in the rhodamine assay using purified HDL and LDL [5] the effect of apolipoprotein (apo)B-depleted serum/plasma, on these lipid probe interactions is more difficult to define. Increased levels of proteins that are scavengers of ROS [11,12] in patients with systemic inflammatory conditions are contained in (apo) B-depleted serum/plasma [13] and can lead to reduction in DOR [14]. However, these biochemical interactions are not present when purified HDL (isolated using ultracentrifugation) is used [14]. We found that the readout using purified HDL highly correlates with the readout using non-purified (apoB-depleted serum/plasma) [14] and a mathematic formula can be used to transform the data (Supplemental Figure 3).

FIGURE LEGENDS

Figure 1

A) The DHR oxidation rate (DOR) was determined as described in Materials and Methods for 50 cryopreserved serum blood bank specimens from healthy subjects. The values represent means of triplicate samples. There was an approximately 10-fold difference between the lowest and highest DOR value (median 10246, IQR 6526-12717 FU/min; range 2069-19325 FU/min).

B) The 50 HDL samples were pooled in groups of five samples (pentads; 0.5 ug of HDL cholesterol from each sample) so that the total amount of HDL cholesterol in each pooled sample (pentad) was 2.5 ug, for a total of 10 pentads. Then the pentads were combined in various combinations and different number (5, 10, 15, 20, 25, 30, 35, 40, 45, 50) of HDL samples so that the total amount of HDL cholesterol at each pooled sample would be 2.5 ug. The DHR oxidation rate (DOR) was determined as described in Materials and Methods. The values represent means of triplicate samples. Using this methodology there was > 3-fold reduction in the variability of determination of DOR (for the same amount of HDL cholesterol) in healthy subjects.

Figure 2

HDL was isolated using PEG precipitation from 3 different groups (A, B, C; each 30 samples) of cryopreserved serum blood bank specimens as described in Methods. The HDL samples in each group were pooled as described in Figure 1. The DHR oxidation rate (DOR) was determined as described in Materials and Methods. The mean DOR of pooled control A (10680 ± 424 FU/min)

was similar to the mean DOR of pooled control B (10290 ± 534 FU/min) and to the mean DOR of pooled control C (9445 ± 1450 FU/min).

Figure 3

A) HDL was isolated from serum of 100 patients with HIV infection (from a different cohort [14]), using ultracentrifugation (purified HDL) and PEG precipitation (non-purified HDL; apoB depleted serum) as previously described [5, 14]. Oxidation of DHR in the presence of these 100 different HDL samples was assessed as described in Methods, using 2.5 µg (cholesterol) of added HDL. The slope of oxidation of each sample (means of quadruplicates) was normalized as ratio to the DOR of a control pool of HDL isolated from serum of 50 healthy control subjects using ultracentrifugation and PEG precipitation as described in Methods. The normalized DOR (nDOR) and correlation coefficient between nDOR in the presence of purified versus non-purified HDL are shown.

B) HDL was isolated from serum of the 93 study subjects with HIV infection, using PEG precipitation (non-purified HDL; apoB depleted serum) and the nDOR was measured as outlined in Figure 3A. Using the formula shown in Figure 3A these data were mathematically transformed to account for the biochemical interactions seen with non-purified HDL. In addition, the nDOR was determined for these 93 samples in the presence of purified HDL (isolated by ultracentrifugation) as outlined in the Methods. The correlation coefficient between the transformed nDOR and the nDOR in the presence of purified HDL is shown.

References

(1) Price TH: *Standards for blood banks and transfusion services.* 25th ed, Bethesda (MD): American Association of Blood Banks; 2008

(2) Boulton F. **Evidence-based criteria for the care and selection of blood donors, with some comments on the relationship to blood supply, and emphasis on the management of donation-induced iron depletion.** *Transfus Med* 2008; **18**:13-27.

(3) American Red Cross [http://www.redcrossblood.org/donating-blood/eligibility-requirements].

(4) Charles-Schoeman C, Khanna D, Furst DE, McMahon M, Reddy ST, Fogelman AM, Paulus HE, Park GS, Gong T, Ansell BJ. **Effects of high-dose atorvastatin on antiinflammatory properties of high density lipoprotein in patients with rheumatoid arthritis: a pilot study.** J Rheumatol 2007; **34**:1459-1464.

(5) Kelesidis T, Currier JS, Huynh D, Meriwether D, Charles-Schoeman C, Reddy ST, Fogelman AM, Navab M, Yang OO. **A Biochemical Fluorometric Method for Assessing the Oxidative Properties of HDL.** *J Lipid Res* 2011; **52**:2341-2351.

(6) Esposito BP, Breuer W, Sirankapracha P, Pootrakul P, Hershko C, Cabantchik ZI. **Labile plasma iron in iron overload: redox activity and susceptibility to chelation.** *Blood* 2003; **102**:2670-2677.

(7) Navab M, Hama SY, Hough GP, Subbanagounder G, Reddy ST, Fogelman AM. **A cell-free assay for detecting HDL that is dysfunctional in preventing the formation of or inactivating oxidized phospholipids**. *J Lipid Res* 2001; **42**:1308-1317.

(8) Kelesidis T, Currier JS, Huynh D, Meriwether D, Charles-Schoeman C, Reddy ST, Fogelman AM, Navab M, Yang OO. **A biochemical fluorometric method for assessing the oxidative properties of HDL.** *J Lipid Res* 2011; 52:2341-2351.

(9) Stocker R, Keaney JF, Jr. **Role of oxidative modifications in atherosclerosis.** *Physiol Rev* 2004; **84**:1381-1478.

(10) Panzenbock U, Kritharides L, Raftery M, Rye KA, Stocker R. **Oxidation of methionine residues to methionine sulfoxides does not decrease potential antiatherogenic properties of apolipoprotein A-I.** *J Biol Chem* 2000; **275**:19536-19544.

(11) Watanabe J, Chou KJ, Liao JC, Miao Y, Meng HH, Ge H, Grijalva V, Hama S, Kozak K, Buga G, Whitelegge JP, Lee TD, Farias-Eisner R, Navab M, Fogelman AM, Reddy ST. **Differential association of hemoglobin with proinflammatory high density lipoproteins in atherogenic/ hyperlipidemic mice. A novel biomarker of atherosclerosis.** *J Biol Chem* 2007; **282**:23698-23707.

(12) Watanabe J, Grijalva V, Hama S, Barbour K, Berger FG, Navab M, Fogelman AM, Reddy ST. **Hemoglobin and its scavenger protein haptoglobin associate with apoA-1-containing particles and influence the inflammatory properties and function of high density lipoprotein.** *J Biol Chem* 2009; **284**:18292-18301.

(13) Charles-Schoeman C, Watanabe J, Lee YY, Furst DE, Amjadi S, Elashoff D, Park G, McMahon M, Paulus HE, Fogelman AM, Reddy ST.**Abnormal function of high-density lipoprotein is associated with poor disease control and an altered protein cargo in rheumatoid arthritis.** *Arthritis Rheum* 2009; **60**:2870-2879.

(14) Kelesidis T, Reddy ST, Huynh D, Meriwether D, Fogelman AM, Navab M, Yang OO. **Effects of lipid-probe interactions in biochemical fluorometric methods that assess HDL redox activity.** *Lipids Health Dis* 2012; **11**:87.

Supplemental Figure 1

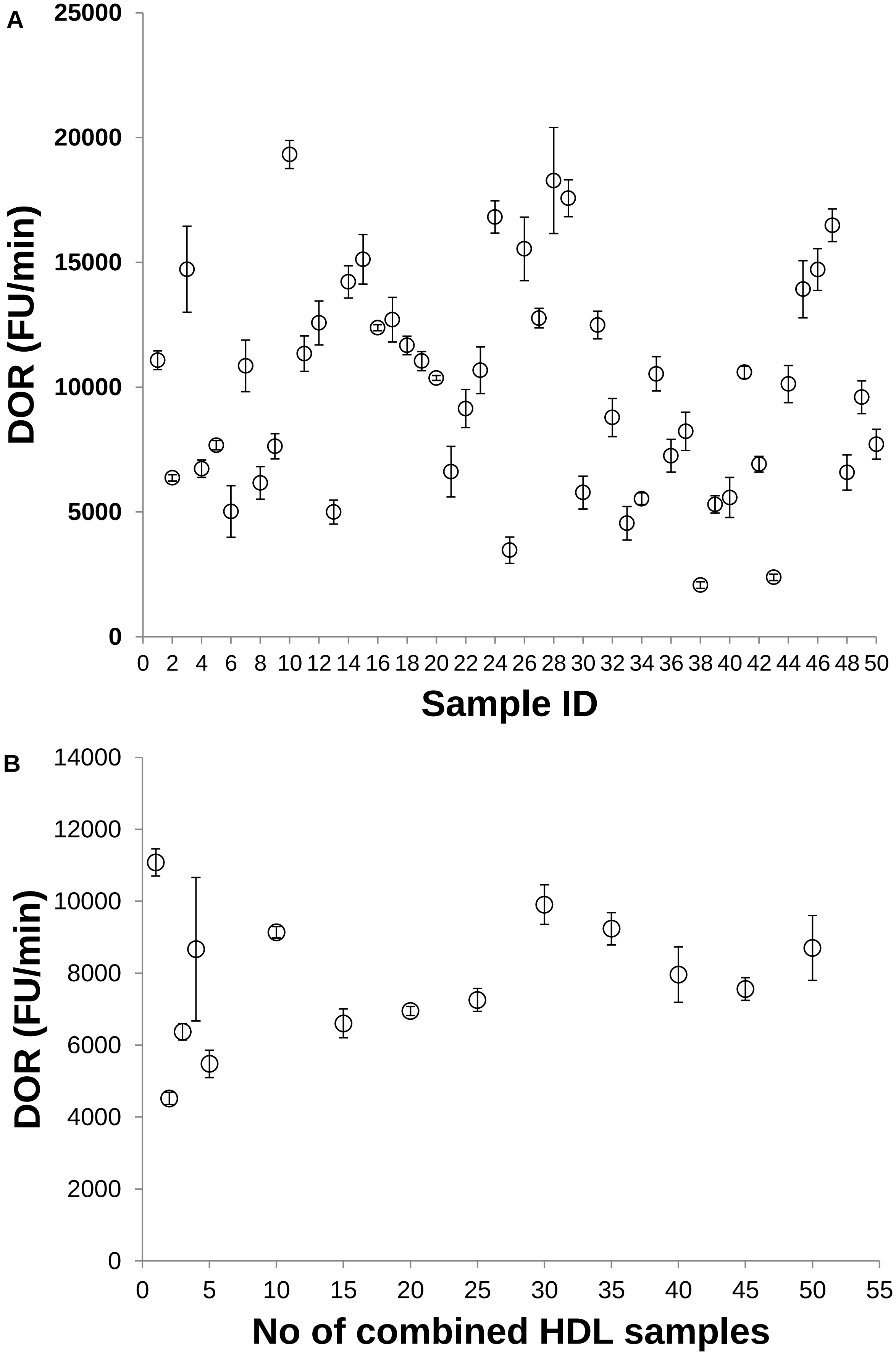

Supplemental Figure 2

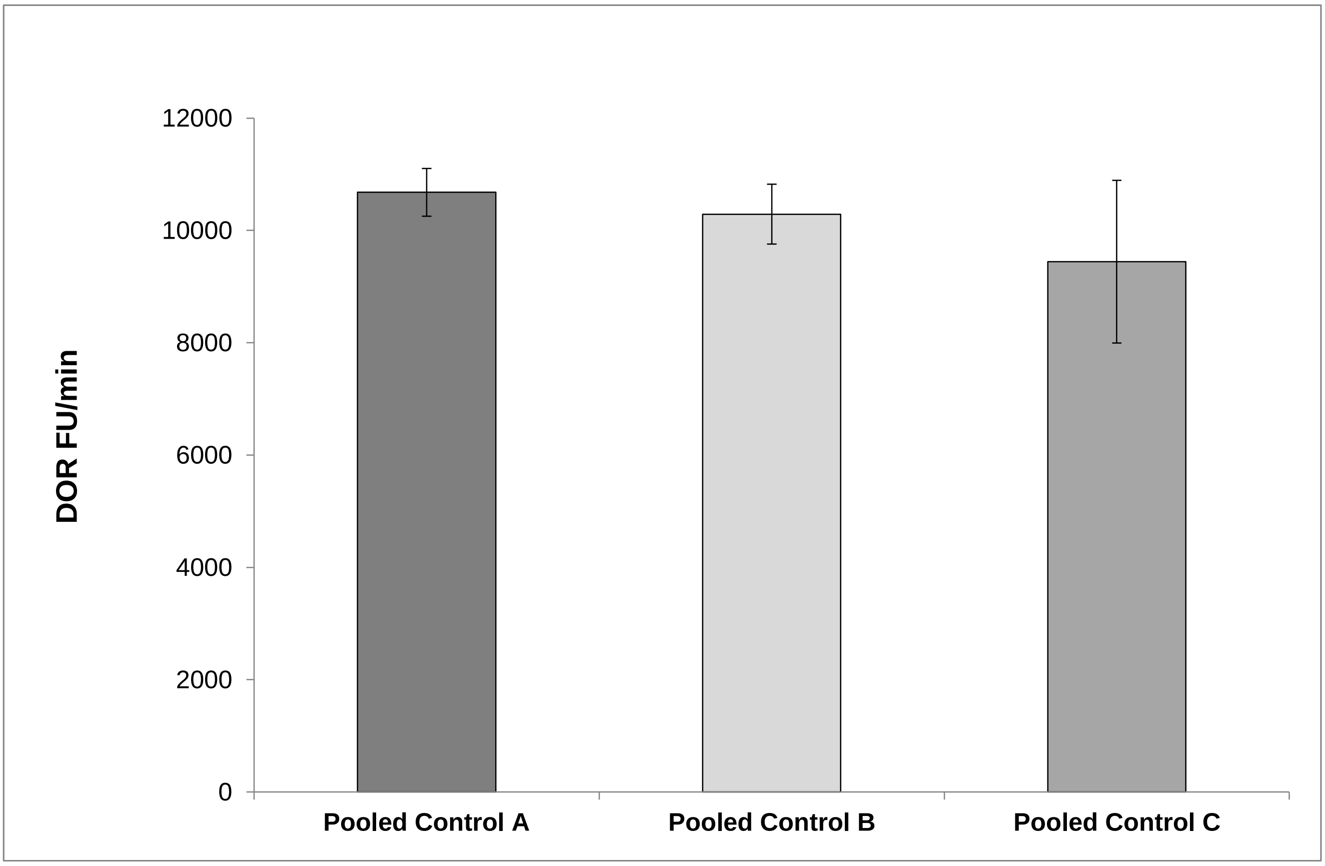

Supplemental Figure 3

A

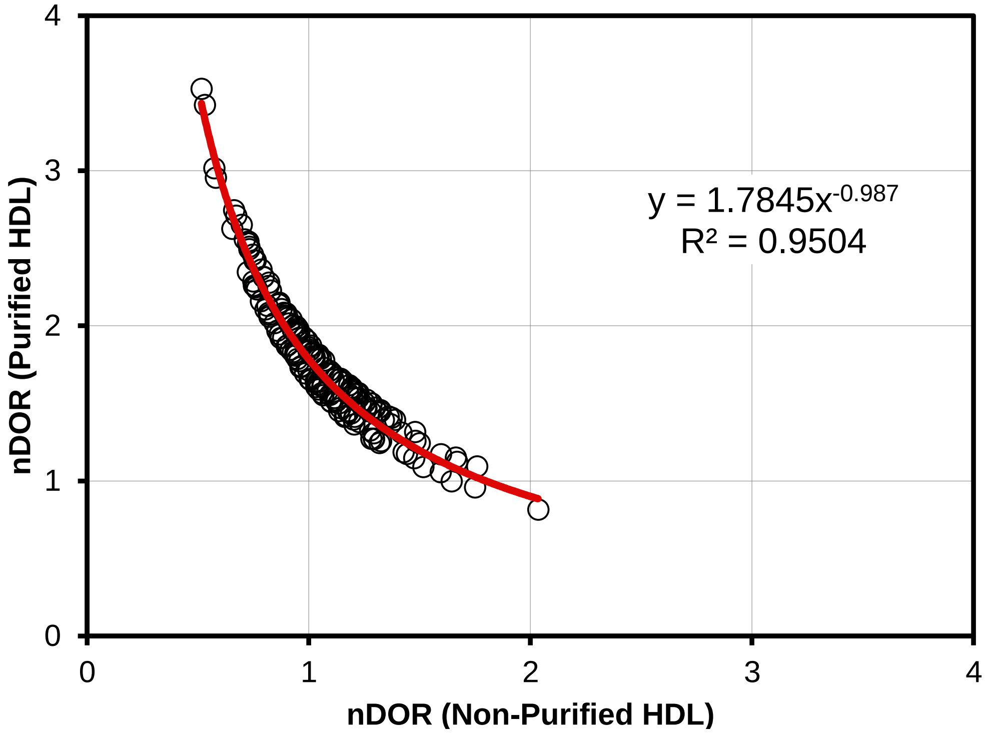

B

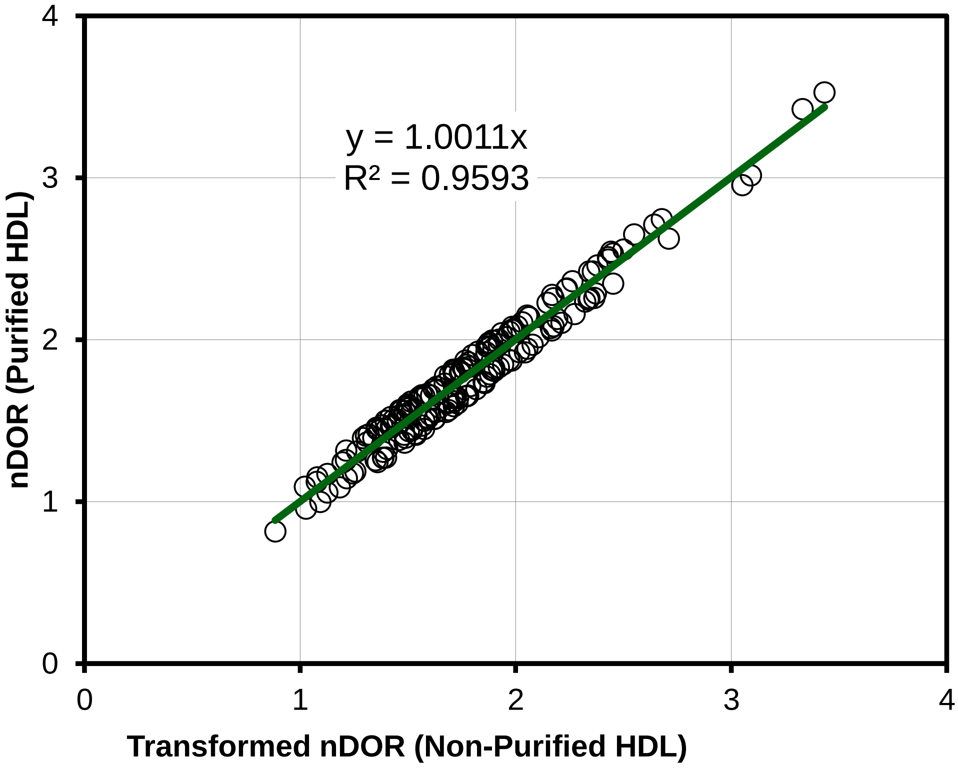

Supplement: Additional file 1 — Supplemental material. [file 1476-511X-12-23-S1.pdf]
